# Our C-Arm-Free Minimally Invasive Technique for Spinal Surgery: The Thoracolumbar and Lumbar Spine—Based on Our Experiences

**DOI:** 10.3390/medicina59122116

**Published:** 2023-12-04

**Authors:** Konstantinos Zygogiannis, Masato Tanaka, Naveen Sake, Shinya Arataki, Yoshihiro Fujiwara, Takuya Taoka, Koji Uotani, Abd El Kader Al Askar, Ioannis Chatzikomninos

**Affiliations:** 1 Department of Orthopaedic Surgery, Okayama Rosai Hospital, Okayama 702-8055, Japan; zygogianniskonstantinos@gmail.com (K.Z.); naveen.sake@gmail.com (N.S.); araoyc@gmail.com (S.A.); fujiwarayoshihiro2004@yahoo.co.jp (Y.F.); taokatakuya@gmail.com (T.T.); coji.uo@gmail.com (K.U.); abdelkader.alaskar@makassedhospital.org (A.E.K.A.A.); 2 Department of Scoliosis and Spine, KAT Hospital, 14561 Athens, Greece; chatzio69@gmail.com

**Keywords:** C-arm free, minimally invasive spine surgery, adult spinal deformity, lateral access spine surgery, oblique lumbar interbody fusion

## Abstract

*Background and Objectives*: The implementation of intraoperative imaging in the procedures performed under the guidance of the same finds its history dating back to the early 1990s. This practice was abandoned due to many deficits and practicality. Later, fluoroscopy-dependent techniques were developed and have been used even in the present time, albeit with several disadvantages. With the recent advancement of several complex surgical techniques, which demand higher accuracy and are in conjunction with the existence of radiation exposure hazard, C-arm-free techniques were introduced. In this review study, we aim to demonstrate the various types of these techniques performed in our hospital. *Materials and Methods*: We have retrospectively analyzed and collected imaging data of C-arm-free, minimally invasive techniques performed in our hospital. The basic steps of the procedures are described, following with a discussion, along with the literature of findings, enlisting the merits and demerits. *Results*: MIS techniques of the thoracolumbar and lumbar spine that do not require the use of the C-arm can offer excellent results with high precision. However, several disadvantages may prevail in certain circumstances such as the navigation accuracy problem where in the possibility of perioperative complications comes a high morbidity rate. *Conclusions*: The accustomedness of performing these techniques requires a steep learning curve. The increase in accuracy and the decrease in radiation exposure in complex spinal surgery can overcome the burden hazards and can prove to be cost-effective.

## 1. Introduction

Over the last few years, with the development of intraoperative O-arm imaging and guidance, the surgical practice of C-arm-free techniques has rapidly expanded. Initially, image-guided spine techniques, which were found to be in practice during the early 1990s, were solely relying on systems that were developed for cranial procedures [1]. In the late 1990s, fluoroscopy-based navigation was introduced but the same showed several limitations, such as exposure to radiation, image distortion, and the lack of an axial view [2]. Even the use of a preoperative computed tomography scan can compromise the accuracy since it is always performed with the patient in a supine position and the majority of spinal interventions are performed with the patient in a prone or lateral position [3]. The accessibility to an O-arm in the operation room empowered us to significantly increase the accuracy by providing live feedback and reducing radiation exposure in comparison to techniques utilizing fluoroscopy [4,5].

On the other hand, adept use of C-arm-free techniques requires a learning curve to be scaled since the operating time in the early stages is often prolonged [6]. Additionally, multilevel procedures may often require more than one O-arm scan, leaving the patient with additional radiation exposure, which is still comparatively less than that which is carried out during the use of a C-arm [7]. Nowadays, the utilization of C-arm-free techniques enables us to perform minimally invasive complex surgical procedures with reduced blood loss, lesser muscle detachment, and higher accuracy even in deformity correction cases, where screw placement may seem to be difficult [8]. Our review paper aims to demonstrate and summarize the surgical examples of C-arm-free techniques for the thoracic and lumbar spine, which are being performed in our hospital.

## 2. Thoracolumbar Spine

### 2.1. Thoracolumbar Anterior Application

#### MIS Corpectomy with a Navigated Expandable Vertebral Cage [9]

**Figure 1:**
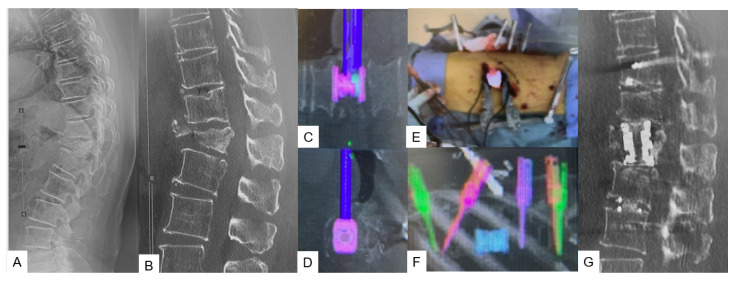
Case 1, 83-year-old female, T12 osteoporotic vertebral fracture, T12 MIS corpectomy. (**A**) Preoperative lateral spine radiogram, (**B**) Preoperative sagittal reconstruction CT, (**C**) Intraoperative coronal navigation image, (**D**) Intraoperative axial navigation image, (**E**) Intraoperative image, (**F**) Intraoperative pedicle screws and cage navigation image, (**G**) Postoperative sagittal reconstruction CT.

A left oblique skin incision of 5 cm, previously verified with a navigated pointer, is most suitable for this procedure as the left side allows a natural anatomical corridor between the aorta and the rest of the intra-abdominal structures. If the corpectomy involves L5 vertebra, the tip of the iliac wing should be resected. During the exposure, it is essential to recognize and isolate the common iliac vessels and the left ureter. The most important point consists of identifying and clipping the ascending lumbar and iliolumbar veins. Before corpectomy is initiated, discectomies at the upper and lower disc levels should be performed. As a next step, the vertebra in the process can be removed by using a combination of navigated osteotome, pituitary forceps, navigated shaver, Kerrison rongeurs, and ring curettes. Simultaneously, a posterior fixation is performed at the desired levels. When the resection is completed, we recommend the use of an expandable vertebral cage measured with a navigated trial (Figure 1C–E). Because real-time expansion is not available with navigation, fluoroscopy can be used for verification if the situation demands it (Figure 1).

### 2.2. Thoracolumbar Posterior Application

#### 2.2.1. Lateral Bendini

**Figure 2:**
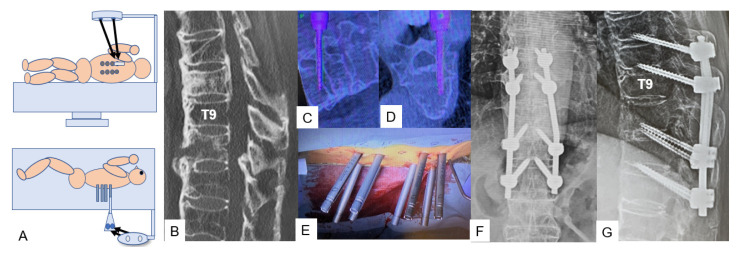
Case 2, 82-year-old male, DISH, T10 fracture, Lateral Bendini. (**A**) Bendini image, (**B**) Preoperative sagittal reconstruction CT, (**C**,**D**) Navigation monitor, (**E**) Intraoperative image, (**F**) Postoperative anteroposterior radiogram, (**G**) Postoperative lateral radiogram.

Following corpectomy or posterior fixation in the lateral decubitus position for DISH fractures, manual rod contouring and insertion can sometimes prove to be challenging. For this technique, a special computer-assisted rod bending system (NuVasive) is used following the percutaneous screw insertion (Figure 2A). Initially, the rod length and the required contouring are measured using the tulip-screw heads as digital markers. Afterwards, the rod is introduced through a special device for the necessary manipulation in both coronal and sagittal planes. Finally, the rod is inserted and placed by adjusting it manually. In this way, the surgeon is able to decrease the screw strain, reduce the operating time, and minimize residual rod overhang (Figure 2).

#### 2.2.2. C-Arm-Free Trauma

**Figure 3:**
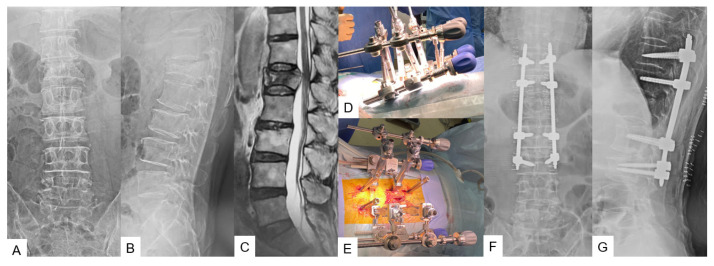
Case 3, 73-year-old male, L1 osteoporotic vertebral fracture, T11-L3 percutaneous posterior reduction. (**A**) Preoperative anteroposterior radiogram, (**B**) Preoperative lateral radiogram, (**C**) Preoperative T2 weighted MR imaging, (**D**,**E**) Intraoperative image, (**F**) Preoperative anteroposterior radiogram, (**G**) Postoperative lateral radiogram.

For posterior fixation in thoracolumbar spine trauma, the patient to be in a prone position is usually indicated. A small mid-line incision above the appropriate spinous process is made for the reference frame attachment followed by O-arm scanning. Next, the pedicle entry points are marked by using a navigated pointer. The pedicle tract is made using an order of navigated instruments, burr, probe, and tapping. Generally, a 2 cm incision is adequate for a single pedicle screw insertion. For the reduction in the fracture, the insertion of a sagittal alignment screw (SAS-Medtronic Solera Longitude II) above and below the fractured vertebra is necessary (Figure 3D,E). The reduction is feasible by using a correction device, which allows distraction with lengthening and angulation maneuvers. Ligamentotaxis achieved through the stretching of the posterior longitudinal ligament plays an important role. If the canal compromise is more than 50% in the CT scan, a rupture should be considered. Finally, the correction should always be assessed intraoperatively with an X-ray (Figure 3).

## 3. Lumbar Spine

### 3.1. Lumbar Anterior Application

#### 3.1.1. OLIF51 [10]

**Figure 4:**
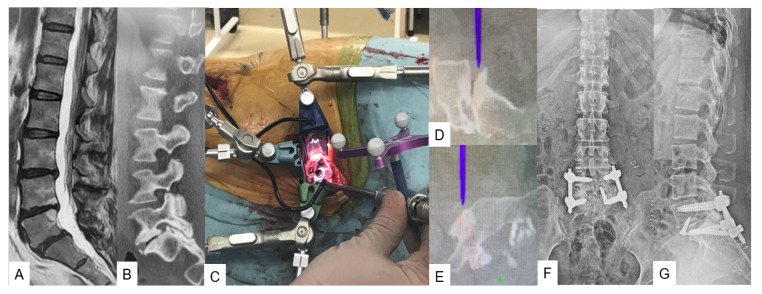
Case 4, 52-year-old female, L5 degenerative spondylolisthesis, L5/S simultaneous OLIF51 and PPS. (**A**) Preoperative T2 weighted MR imaging, (**B**) Preoperative CT, (**C**) Intraoperative image, (**D**,**E**) Navigation monitor, (**F**) Preoperative anteroposterior radiogram, (**G**) Postoperative lateral radiogram.

The patient in the lateral decubitus, left-sided surgical approach is necessary for this operation. The reference frame is placed at the sacroiliac joint as it is considered to be very stable. A 4 cm oblique skin incision, previously verified with a navigated pin-pointer, is usually sufficient. The incision should be at the center and parallel to the L5-S1 discs, while at the same time 4 cm medial to the anterior superior iliac crest and parallel to the pelvis. During the surgical exposure, the abdominal muscles, which include the external oblique, internal oblique, and transverse abdominis along with their fascia, should be divided along with the muscle fibers and not cut. The use of a monopolar cautery is prohibited due to the potential risk of injury to the ilioinguinal and iliohypogastric nerves. While being at the retroperitoneal space, blunt finger dissection should be used down to the internal abdominal wall to reach the psoas muscle. After exposing the promontorium, the left iliac vessels should be retracted upwards while preserving the left ureter to be undamaged. Once the L5-S1 discs are fully exposed, the disc space preparations should be commenced followed by cage size trials (Figure 4C–E). Finally, an intraoperative X-ray should be carried out to confirm the cage size and position (Figure 4). The clinical and radiological results are summarized in Table 1 and Table 2.

#### 3.1.2. Lateral Osteotomy [11]

**Figure 5:**
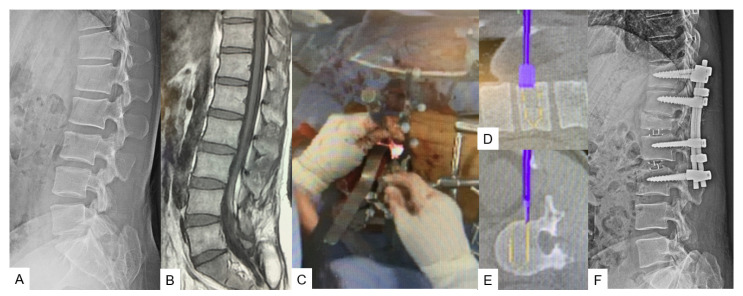
Case 5, 33-year-old male, Tethered cord syndrome, L1 shortening osteotomy with lateral osteotomy. (**A**) Preoperative lateral radiogram, (**B**) Preoperative T1 weighted MR imaging, (**C**) Intraoperative image, (**D**,**E**) Navigation monitor, (**F**) Preoperative lateral radiogram.

For this procedure, the patient is placed in a right lateral decubitus position in the same way as in OLIF with the table bent approximately 15 degrees in convex. After an oblique 5 cm skin incision is marked with a navigated pin-pointer, the fat is dissected until the abdominal musculature is reached. Then, the external oblique, internal oblique, and transverse abdominis muscles are divided along the direction of the muscle fibers. Blunt finger dissection should be performed to create an adequate working channel through the retroperitoneal area, until the psoas muscle can be visualized. When the disc is fully revealed the process of osteotomy can be initiated. The key to successful correction of spinal deformity lies in sufficient release of contralateral tissue and complete resection of the annulus followed by precise cage placement. A navigated osteotome is utilized to break the fused mass in the contralateral side followed by usage of navigated Cobb, shavers, ring curettes, and pituitary forceps (Figure 5C–E). The required length and height of the cage are measured through the live feedback on the monitor. Usually, this operation is the first stage of a two-stage correction for adult spine deformity. Osteotomies and OLIF can be performed on multiple levels to achieve correction in the sagittal and coronal plane, thereby achieving higher fusion rates (Figure 5).

### 3.2. Lumbar Posterior Application

#### 3.2.1. Adult Spinal Deformity [12]

**Figure 6:**
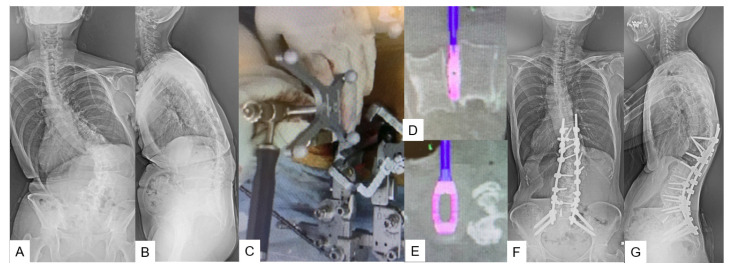
Case 6, 73-year-old female, Adult spinal deformity, L1-S1 OLIF and T10-pelvis posterior fusion. (**A**) Preoperative posteroanterior radiogram, (**B**) Preoperative lateral radiogram, (**C**) Intraoperative image, (**D**,**E**) Navigation monitor, (**F**) Preoperative posteroanterior radiogram, (**G**) Postoperative lateral radiogram.

C-arm-free navigated correction for adult spinal deformity can be a two-stage or one-stage surgery. For this technique, a two-stage correction is ideally preferred. As mentioned in Section 3.2.2, multiple-level lateral C-arm-free osteotomies with cage placement can be performed during the first stage (Figure 6C–E). Then, the amount of correction achieved is assessed through plain radiographs and a CT scan. During the second stage, C-arm-free percutaneous screw fixation is performed, if there is no need for open surgical osteotomy. Additional correction with this technique can be achieved by using in situ benders, distraction–compression, and vertebral derotation. The contralateral tissue release made during the first stage plays an important role in the final result as the curve is less stiff. Finally, after the rod insertion, the final amount of correction should be checked with intraoperative X-rays (Figure 6). The clinical and radiological results of C-arm-free navigated correction for adult spinal deformity are summarized in Table 3.

#### 3.2.2. C-Arm-Free Biopsy [13]

**Figure 7:**
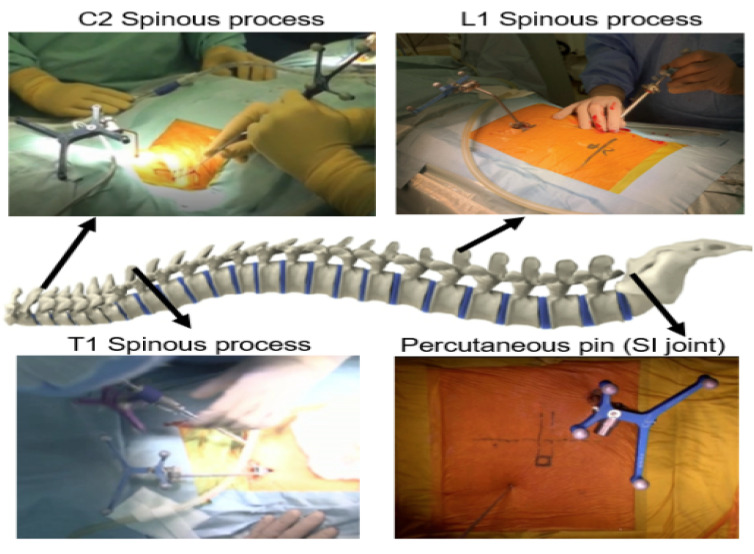
Position of reference frame for C-arm-free biopsy (from reference [13]).

A prone position is best suited for MIS-navigated biopsy. We recommend the following reference frame position for each anatomical region (Figure 7):

1. C2 spinous process for upper cervical spine.

2. C7 and T1 for cervicothoracic junction.

3. T12 and L1 for thoracolumbar junction.

4. Sacroiliac joint for lumbar and pelvic region.

With a skin incision of approximately 5 mm, the cortex of the targeted pedicle is penetrated with the navigated high-speed burr or navigated awl. A navigated probe can be used to reach the targeted lesion. Afterward, a 5 mm tube is inserted and a sample can be taken by using micro pituitary forceps. The merit of navigation in this case consists of the ability to collect a sample with precision by using a navigation check to avoid false negative biopsy results. The special non-navigated tool penetrates only 8 mm beyond the tube. Thus, this maintains safety.

#### 3.2.3. C-Arm-Free Endoscopic Technique [14]

**Figure 8:**
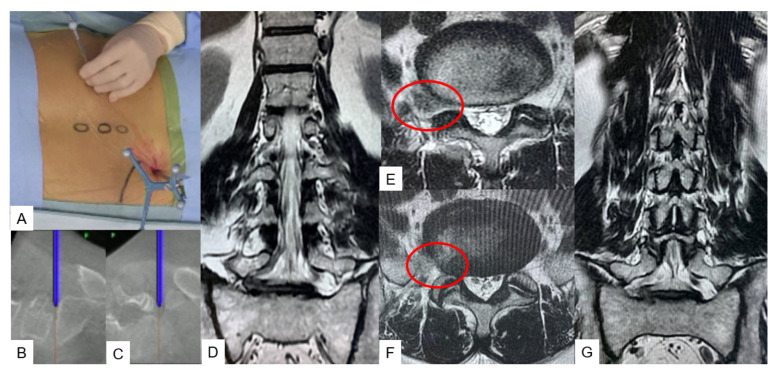
Case 7, 55 M, right L5/S foraminal disc herniation, transforaminal discectomy with navigation, (**A**) Intraoperative image, (**B**) Sagittal navigation image, (**C**) Coronal navigation image, (**D**) Preoperative coronal T2 weighted MR imaging, (**E**) Preoperative axial T2 weighted MR imaging, (**F**) Postoperative axial T2 weighted MR imaging, (**G**) Postoperative coronal T2 weighted MR imaging.

A prone position is suitable for this technique as preferred in all discectomies. To avoid any mechanical obstacles during the surgical procedure, the reference frame is placed into the contralateral sacroiliac joint, followed by an O-arm scanning. An optimal incision point is verified with a navigated pin-pointer. Then, the subcutaneous fat and the muscles are dissected to permit the insertion of the navigated dilators (Figure 8A–C). Once again, the level and the adjacent anatomical structures are verified with a navigated probe. The bone resection is performed using a high-speed burr. Following this, all the necessary sequential actions are performed for nerve root decompression. In atypical cases presenting with a far lateral disc herniation, hard disc, or osteophyte, this technique enables us to continuously verify the exact position of the instruments, making the decompression easier and more precise (Figure 8).

## 4. Discussion

Multiple anatomical approaches have been proposed to access the thoracolumbar and lumbar spine due to the convergence of multiple tissue planes as well as abdominal structures [15]. Characteristics such as the presence of great vessels, risk of bowel injury, patient’s age, and biomechanics, especially regarding the L5 vertebra, where the shearing and compressive forces are high, are often related to higher complication rates [16,17]. Therefore, techniques such as C-arm-free corpectomy by using a navigated expandable cage can offer a reliable surgical treatment [9]. Navigation makes corpectomy feasible while performed with a small incision of 5 cm. The merit of this technique lies in precise cage placement. Cage positioning can prove to be challenging after a corpectomy due to the lordotic angle between the upper and lower segments, particularly between the L4 vertebra and sacrum [17]. C-arm imaging alone can provide sufficient intraoperative information for the cage position; however, by using the navigated expandable cage, the same is possible through live feedback trialing, which sometimes gives us the capability of inserting a larger cage. In addition, MIS corpectomy can offer significantly less blood loss and early mobilization. Yu et al., in a recent study, reported that the postoperative outcomes were comparable between the surgeries performed with the use of an O-arm and C-arm [18]. However, the cage is not navigated in their study. With the usage of a navigated expandable cage, the cage is inserted more precisely [9]. On the contrary, an intraoperative complication for this approach sometimes can prove to be devastating. Siasios et al., in a review, reported that the mortality incidence from a bowel injury can reach 12.9% [19]. Additionally, C-arm-free corpectomy is not considered out of the limitation of possible perioperative complications and also the cost is higher. Thus, with a broader perspective, both aspects of the same coin should be considered.

Various techniques have been proposed to increase the screw pull-out strength in severe osteoporotic cases, such as augmented screw fixation [20,21]. Although they provide certain stability and outcomes [22], in case there is a need for revision surgery, the screw replacement might be a little challenging due to the presence of the cement. Bendini is a spinal rod bending system that can provide intraoperative patient-specific rods. Manual rod contouring is a major step in deformity correction and percutaneous screw fixation; in such cases, inadequate rod contouring can result in poorer results and prolonged surgery time. By forcing the rod into the screw tulip, the pull-out forces can increase significantly [23,24]; the merit of this technique lies in the fact that the rod is precisely contoured based on the pedicle screw placement, which is often asymmetrical [25]. Moreover, the required correction in the sagittal and coronal plane can be expressed in the rod by setting the necessary information in the system. However, the rod still must be inserted manually, so even in cases where the patient has a high BMI, the insertion may prove laborious.

Another application of the C-arm-free technique in the thoracolumbar junction is the treatment of trauma cases. The main principles advocating for this procedure are to provide stability and achieve bone fusion and sufficient alignment. Minimally invasive procedures have been proposed to overcome postoperative complications of open surgery [26]. Issues like infection rates, wound healing, and chronic back pain often arise from open surgery since the muscle detachment and the dead space are wider [27]. Defino et al., in a retrospective study, suggested that the overall complication rate of minimally invasive surgery is 2.1%, significantly lesser compared to the open surgery, which is 9.4% [28]. By practicing C-arm-free techniques for trauma cases, the surgeon is able to uphold those benefits without exposure to radiation and operate with high precision [29,30]. However, for hospitals that receive a large number of trauma patients, this procedure may not be cost-effective.

For anterior lumbar application of C-arm-free techniques, OLIF51 is practically a laterally positioned retroperitoneal anterior lumbar interbody fusion (ALIF) procedure, which has emerged as an effective fusion technique for adult spinal deformity cases. This approach aims to achieve bony stability, improve alignment, and indirectly decompress the neural elements at the L5-S1 level. In conjunction with OLIF25, careful consideration of indications and preoperative anatomical investigations is crucial for a successful OLIF51 surgery [31]. Woods et al., in a retrospective cohort study of 137 patients, reported that the fusion rate for the L5-S1 OLIF procedure was 97.9%. However, the overall complication rate was 11.7%, with the most common complication being subsidence (4.4%), followed by postoperative ileus (2.9%) and vascular injury (2.9%) [32]. In another study conducted at our hospital, we retrospectively analyzed 54 patients with a 2-year follow up, all of whom underwent corrective surgery for adult spinal deformity. The first group underwent transverse lumbar interbody fusion for L5-S1, while the second group underwent the OLIF procedure. We reported that there were no significant clinically varying outcomes between the two groups. However, radiographically, the OLIF resulted in better L5-S1 lordosis [33].

Adult spinal deformity is a frequently occurring disorder that without prompt treatment can result in chronic back pain, poor posture, and disability. Recent studies suggest that conservative treatment in an established deformity case offers poor results [34]. Nevertheless, surgical treatment comes with a high rate of complications such as a high morbidity rate, surgical site infection, proximal junctional failure, and pseudoarthrosis [35,36]. Studies suggest that overall, in some cases, complication rates can reach up to 70% and morbidity to 2.4% [34,37]. Mummameni et al., in a recent study, reported that the incidence rate of neurological complications in patients who had undergone pedicle subtraction osteotomy for spine deformity was 14.9%, while the same in patients who had undergone multilevel OLIF was 2.9% [37]. By performing C-arm-free lateral osteotomies through a small incision, the surgeon is able to reduce the complication rates and increase accuracy. The contralateral release can prove to be demanding due to the presence of great vessels and important intra-abdominal structures. By using a navigated blunt Cobb and a navigated osteotome to cut the fused mass, the surgeon is able to minimize the risk of vessel injury [11]. On the contrary, this technique holds some disadvantages. The risk of endplate fracture and the compromise of accuracy, if the reference frame is moved, is always present. The final stage of correction for ASD can include percutaneous pedicle screw fixation to reduce the complication rates even further [38]. Finally, precision is not only necessary for screw insertion and deformity correction, but also for biopsy and disc herniations. High-precision tissue sample collection can not only lead to an accurate diagnosis but also avoidance of neoplastic cell dissemination [13]. While regarding disc herniation, in the presence of a demanding nerve root decompression, an extended bone resection can lead to instability, back pain, and finally instrumented posterior fixation [39]. The recent literature suggests that MIS techniques offer adequate decompression [40] but with the addition of O-arm techniques, the adequacy of decompression can be verified intraoperatively. The drawback of this technique lies in the fact that accuracy can be lost if the reference frame is moved, so handling with the utmost care and multiple checks of the navigation guided setup should be conducted during the operation [41]. Advantages and disadvantages of the C-arm-free technique are summarized in Table 4.

## 5. Conclusions

C-arm-free techniques can offer reliable and accurate results in all aspects of thoracic and lumbar spine surgical techniques. By increasing accuracy and reducing radiation exposure, the patient and the surgeon are able to obtain certain benefits. However, its cost-effectiveness varies, as not all hospitals own an O-arm or a navigation system; even so, the assets prevail.

## Figures and Tables

**Table 1:** Patient demographic (D).

Patients (54)	Female (50), Male (4)
Age (years)	71.5 ± 6.2
BMI (kg/m^2^)	22.9 ± 4.1
Follow-up period (months)	29.2 ± 8.4

**Table 2:** The clinical and radiological results of OLIF51 and TLIF51.

	OLIF51	TLIF51	
Patients	Men (0), Women (13)	Men (4), Women (3)	0.243
Age (years)	74.6 ± 3.2	70.5 ± 6.6	0.023
BMI (kg/m^2^)	22.7 ± 3.7	23.0 ± 4.2	0.715
Postoperative L5-S1 angle gain (°)	9.4 ± 4.7	1.6 ± 5.1	0.0001
Postoperative L5-S1 height gain (mm)	4.2 ± 2.9	0.8 ± 1.9	0.0002
Reoperation	2	8	0.570

**Table 3:** The clinical and radiological results of C-arm-free navigated correction for adult spinal deformity (F).

	Preoperative Value	Postoperative Value	*p* Value
ODI (%)	46.0 ± 10.4	30.5 ± 18.9	*p* < 0.01
VAS (mm)	52.9 ± 7.3	31.2 ± 6.9	*p* < 0.01
SVA (mm)	96.5 ± 55.9	24.1 ± 39.0	*p* < 0.01
PT (degree)	34.5 ± 11.0	17.1 ± 10.3	*p* < 0.01
LL (degree)	13.3 ± 18.6	49.1 ± 9.7	*p* < 0.01
PI—LL (degree)	39.3 ± 22.1	2.4 ± 12.6	*p* < 0.01

**Table 4:** Advantages and disadvantages of C-arm-free technique.

Advantages	Disadvantages
Less radiation hazard	Learning curve
Guidance to cage placement	Need of C-arm to verify dynamic changes
Contralateral release	Cost-effectiveness

## Data Availability

No new data were created or analyzed in this study. Data sharing is not applicable to this article.

## References

[1] Helm P.A., Teichman R., Hartmann S.L., Simon D. (2015). Spinal Navigation and Imaging: History, Trends, and Future. IEEE Trans. Med. Imaging.

[2] Rawicki N., Dowdell J.E., Sandhu H.S. (2021). Current state of navigation in spine surgery. Ann. Transl. Med..

[3] Fan X., Mirza S.K., Li C., Evans L.T., Ji S., Paulsen K.D. (2022). Accuracy of Stereovision-Updated Versus Preoperative CT-Based Image Guidance in Multilevel Lumbar Pedicle Screw Placement: A Cadaveric Swine Study. JB JS Open Access.

[4] Rampersaud Y.R., Foley K.T., Shen A.C., Williams S., Solomito M. (2000). Radiation exposure to the spine surgeon during fluoroscopically assisted pedicle screw insertion. Spine.

[5] Mroz T.E., Abdullah K.G., Steinmetz M.P., Klineberg E.O., Lieberman I.H. (2011). Radiation exposure to the surgeon during percutaneous pedicle screw placement. J. Spinal Disord. Tech..

[6] Shuman W.H., Valliani A.A., Chapman E.K., Martini M.L., Neifert S.N., Baron R.B., Schupper A.J., Steinberger J.M., Caridi J.M. (2022). Intraoperative Navigation in Spine Surgery: Effects on Complications and Reoperations. World Neurosurg..

[7] Bratschitsch G., Leitner L., Stücklschweiger G., Guss H., Sadoghi P., Puchwein P., Leithner A., Radl R. (2019). Radiation Exposure of Patient and Operating Room Personnel by Fluoroscopy and Navigation during Spinal Surgery. Sci. Rep..

[8] Van de Kelft E., Costa F., Van der Planken D., Schils F. (2012). A prospective multicenter registry on the accuracy of pedicle screw placement in the thoracic, lumbar, and sacral levels with the use of the O-arm imaging system and stealthstation navigation. Spine.

[9] Yamauchi T., Jaiswal A., Tanaka M., Fujiwara Y., Oda Y., Arataki S., Misawa H. (2021). Minimally Invasive L5 Corpectomy with Navigated Expandable Vertebral Cage: A Technical Note. Brain Sci..

[10] Tanaka M., Singh M., Fujiwara Y., Uotani K., Oda Y., Arataki S., Yamauchi T., Takigawa T., Ito Y. (2022). Comparison of Navigated Expandable Vertebral Cage with Conventional Expandable Vertebral Cage for Minimally Invasive Lumbar/Thoracolumbar Corpectomy. Medicina.

[11] Tanaka M., Uotani K., Fujiwara Y., Yamane K., Sonawane S., Arataki S., Yamauchi T. (2021). Navigated Lateral Osteotomy for Adult Spinal Deformity: A Technical Note. World Neurosurg..

[12] Kumar B.S., Tanaka M., Arataki S., Fujiwara Y., Mushtaq M., Taoka T., Zygogiannnis K., Ruparel S. (2023). Lateral access minimally invasive spine surgery in adult spinal deformity. J. Orthop..

[13] Tanaka M., Sonawane S., Uotani K., Fujiwara Y., Sessumpun K., Yamauchi T., Sugihara S. (2021). Percutaneous C-Arm Free O-Arm Navigated Biopsy for Spinal Pathologies: A Technical Note. Diagnostics.

[14] Tanaka M., Arataki S., Mehta R., Tsai T.T., Fujiwara Y., Uotani K., Yamauchi T. (2022). Transtubular Endoscopic Posterolateral Decompression for L5-S1 Lumbar Lateral Disc Herniation. J. Vis. Exp..

[15] Xu D.S., Walker C.T., Farber S.H., Godzik J., Gandhi S.V., Koffie R.M., Turner J.D., Uribe J.S. (2022). Surgical anatomy of minimally invasive lateral approaches to the thoracolumbar junction. J. Neurosurg. Spine.

[16] Quraishi N.A., Konig M., Booker S.J., Shafafy M., Boszczyk B.M., Grevitt M.P., Mehdian H., Webb J.K. (2013). Access related complications in anterior lumbar surgery performed by spinal surgeons. Eur. Spine J..

[17] Vazan M., Ryang Y.M., Gerhardt J., Zibold F., Janssen I., Ringel F., Gempt J., Meyer B. (2017). L5 corpectomy-the lumbosacral segmental geometry and clinical outcome-a consecutive series of 14 patients and review of the literature. Acta Neurochir..

[18] Yu J., Fridley J., Gokaslan Z., Telfeian A., Oyelese A.A. (2019). Minimally invasive thoracolumbar corpectomy and stabilization for unstable burst fractures using intraoperative computed tomography and computer-assisted spinal navigation. World Neurosurg..

[19] Siasios I., Vakharia K., Khan A., Meyers J.E., Yavorek S., Pollina J., Dimopoulos V. (2018). Bowel injury in lumbar spine surgery: A review of the literature. J. Spine Surg..

[20] Shea T.M., Laun J., Gonzalez-Blohm S.A., Doulgeris J.J., Lee W.E., Aghayev K., Vrionis F.D. (2014). Designs and techniques that improve the pullout strength of pedicle screws in osteoporotic vertebrae: Current status. Biomed Res. Int..

[21] Tomé-Bermejo F., Piñera A.R., Alvarez-Galovich L. (2017). Osteoporosis and the management of spinal degenerative disease (I). Arch. Bone Jt. Surg..

[22] Son H.J., Choi S.H., Heo D.R., Kook I., Lee M.K., Ahn H.S., Kang C.N. (2021). Outcomes of the use of cement-augmented cannulated pedicle screws in lumbar spinal fusion. Spine J..

[23] Sumiya S., Fukushima K., Kurosa Y., Hirai T., Inose H., Yoshii T., Okawa A. (2021). Comparative analysis of clinical factors associated with pedicle screw pull-out during or immediately after surgery between intraoperative cone-beam computed tomography and postoperative computed tomography. BMC Musculoskelet Disord..

[24] Matthews P.G.M., Cadman J., Tomka J., Dabirrahmani D., Appleyard R., Kam A. (2020). Pullout force of minimally invasive surgical and open pedicle screws-a biomechanical cadaveric study. J. Spine Surg..

[25] Tohmeh A.G., Isaacs R.E., Dooley Z.A., Turner A.W.L. (2014). Long Construct Pedicle Screw Reduction and Residual Forces are Decreased Using a Computer-assisted Rod Bending System. J. Spine Neurosurg..

[26] Tinelli M., Töpfer F., Kreinest M., Matschke S., Grützner P.A., Suda A.J. (2018). Minimally invasive reduction and percutaneous posterior fixation of one-level traumatic thoraco-lumbar and lumbar spine fractures. Eur. J. Orthop. Surg. Traumatol..

[27] Ni W.F., Huang Y.X., Chi Y.L., Xu H.Z., Lin Y., Wang X.Y., Huang Q.S., Mao F.M. (2010). Percutaneous pedicle screw fixation for neurologic intact thoracolumbar burst fractures. J. Spinal Disord. Tech..

[28] Defino H.L.A., Costa H.R.T., Nunes A.A., Barbosa M.N., Romero V. (2019). Open versus minimally invasive percutaneous surgery for surgical treatment of thoracolumbar spine fractures- a multicenter randomized controlled trial: Study protocol. BMC Musculoskelet. Disord..

[29] Masse F.X. (2009). Dosimetry Report of the Medtronic O-Arm System.

[30] Jones D.P.G., Robertson P.A., Lunt B., Phys M., Jackson S.A. (2000). Radiation exposure during fluoroscopically assisted pedicle screw insertion in the lumbar spine. Spine.

[31] Orita S., Inage K., Furuya T., Koda M., Aoki Y., Kubota G., Nakamura J., Shiga Y., Matsuura Y., Maki S. (2017). Oblique Lateral Interbody Fusion (OLIF): Indications and techniques. Oper. Tech. Orthop..

[32] Woods K.R., Billys J.B., Hynes R.A. (2017). Technical description of oblique lateral interbody fusion at L1-L5 (OLIF25) and at L5-S1 (OLIF 51) and evaluation of complication and fusion rates. Spine J. Off. J. North Am. Spine Soc..

[33] Smith J.S., Lafage V., Shaffrey C.I., Schwab F., Lafage R., Hostin R., O’brien M., Boachie-Adjei O., Akbarnia B.A., Mundis G.M. (2016). Outcomes of operative and nonoperative treatment for adult spinal deformity: A prospective, multicenter, propensity-matched cohort assessment with minimum 2-year follow-up. Neurosurgery.

[34] Akıntürk N., Zileli M., Yaman O. (2022). Complications of adult spinal deformity surgery: A literature review. J. Craniovertebr. Junction Spine..

[35] Dinizo M., Dolgalev I., Passias P.G., Errico T.J., Raman T. (2021). Complications After Adult Spinal Deformity Surgeries: All Are Not Created Equal. Int. J. Spine Surg..

[36] Lau D., Osorio J.A., Deviren V., Ames C.P. (2018). The relationship of older age and perioperative outcomes following thoracolumbar three-column osteotomy for adult spinal deformity: An analysis of 300 consecutive cases. J. Neurosurg. Spine.

[37] Mummaneni P.V., Park P., Shaffrey C.I., Wang M.Y., Uribe J.S., Fessler R.G., Chou D., Kanter A.S., Okonkwo D.O., Mundis G.M. (2019). The MISDEF2 algorithm: An updated algorithm for patient selection in minimally invasive deformity surgery. J. Neurosurg. Spine.

[38] Lykissas M.G., Giannoulis D. (2018). Minimally invasive spine surgery for degenerative spine disease and deformity correction: A literature review. Ann. Transl. Med..

[39] Lee Y.P., Sclafani J. (2013). Lumbar iatrogenic spinal instability. Semin. Spine Surg..

[40] Wong K.W., Ho C.H., Yu T.C., Wu C.D., Tsang Y.S. (2018). Clinical Outcome of Minimally Invasive Decompression Without Discectomy in Contained Foraminal Disc Herniation: A Single-Center Study. World Neurosurg..

[41] Guha D., Jakubovic R., Gupta S., Fehlings M.G., Mainprize T.G., Yee A., Yang V.X.D. (2019). Intraoperative Error Propagation in 3-Dimensional Spinal Navigation From Nonsegmental Registration: A Prospective Cadaveric and Clinical Study. Global Spine J..

